# Cross-Layer Design for Energy-Efficient Reliable Multi-Path Transmission in Event-Driven Wireless Sensor Networks

**DOI:** 10.3390/s23146520

**Published:** 2023-07-19

**Authors:** Huihui Xu, Xiaobing Yuan

**Affiliations:** 1 Science and Technology on Micro-System Laboratory, Shanghai Institute of Microsystem and Information Technology, Chinese Academy of Sciences, Shanghai 201800, China; xuhuihui@mail.sim.ac.cn; 2 University of Chinese Academy of Sciences, Beijing 100049, China

**Keywords:** multi-event-driven, multi-path transmission, cross-layer design, energy efficiency maximization, dual decomposition

## Abstract

In event-driven wireless sensor networks (WSNs), a reliable, efficient, and scalable routing solution is required for the reliable delivery of sensory data to the base station (BS). However, existing routing algorithms rarely address the issue of energy efficiency under multi-path conflicts for multi-event-driven scenarios. In order to maximize energy efficiency while maintaining a manageable conflict probability, this paper investigates a cross-layer design of routing and power control for multi-event-driven WSNs. We first develop a mathematical characterization of the conflict probability in multi-path routing, and we then formulate the energy efficiency maximization problem as a non-convex combinatorial fractional optimization problem subject to a maximum conflict probability constraint. By utilizing non-linear fractional programming and dual decomposition, an iterative search algorithm was used to obtain near-optimal power allocation and routing solutions. Extensive results demonstrate that our proposed algorithm achieved a gain of 9.09% to 35.05% in energy efficiency compared to other routing algorithms, thus indicating that our proposed algorithm can avoid unnecessary control overhead from multi-path conflicts with a lower conflict probability and can ensure maximum energy efficiency through routing and power control design.

## 1. Introduction

WSNs typically consist of a series of distributed sensor nodes that generate data in response to event triggering and forward it to the BS via multi-hop transmission. Reliable and energy-efficient transmission are two of the most critical issues in the design of routing algorithms for wireless networks, especially for dense networks with stringent energy constraints.

For the former, the random selection of intermediate nodes reduces the stability of the route due to the uncertainty of node behavior, thereby jeopardizing the reliability of data transmission and thus reducing the performance of the network in terms of the packet delivery rate (PDR) [1]. When there is an intermediate node interruption in the route, it may cause multi-hop communication to fail. The nodes must rely on route discovery cycles or timeouts to reestablish routes [2]. This not only leads to a proliferation of network-wide routing requests that consume significant network resources, but also increases packet transmission delays. Therefore, in order to establish stable routing and maintain continuous traffic flow, it is necessary to evaluate the ability and reliability of nodes in relaying transmissions to make informed routing decisions. In recent years, many reliable routing algorithms have been proposed for wireless networks [3,4,5,6]. These algorithms improve the reliability of end-to-end transmission by considering the channel state information and the collaboration power information of the nodes to minimize the outage probability.

However, these algorithms have poor scalability due to not considering interference conflicts between multiple paths. As the number of trigger events increases, interference conflicts between routes increase, which may affect the overall performance of the network and prevent the network from achieving the desired performance improvement. Specifically, when the network uses a carrier sense multiple access (CSMA)-based MAC protocol to eliminate contention and retransmission caused by conflicts, the increased probability of inter-path conflict increases the chance of interaction between nodes, which not only brings more interaction overhead, but also reduces transmission concurrency and thus compromises transmission efficiency. Therefore, the conflict probability between multiple paths is also a major factor to be considered in the extension to multi-event-driven routing algorithms.

In addition, due to the explosive demand for data services and energy-intensive wireless devices, energy efficiency is another critical issue in the design of routing algorithms and is of great importance for energy-constrained WSNs. Many energy-efficiency-oriented studies incorporate power control techniques to reduce network energy consumption [7,8,9]. In addition, there are several studies that achieved power reduction in routing designs via multi-hop short-range data communication [10,11,12]. However, paths with a large number of relays imply low throughput. The limited energy resources of the sensor nodes force a trade-off between throughput and energy consumption when determining the route to the BS [13]. Energy efficiency, defined in bits/joules, has been increasingly accepted as an attractive metric for assessing the performance of communication systems [14,15]. Therefore, studying the energy efficiency of data transmission is particularly important for the design of future wireless communication systems.

Overall, the study of how to improve transmission reliability within an acceptable conflict probability and how to improve the energy efficiency of the network are important performance requirements in many application scenarios. Especially in multi-event-driven WSNs, the presence of multi-path conflicts not only introduces more interaction overhead, but also reduces transmission concurrency, thus significantly affecting the performance of energy efficiency. To address this problem in multi-event-driven scenarios, this paper proposes a cross-layer optimization problem for joint routing and power control that maximizes energy efficiency within an acceptable conflict probability. The main contributions of the paper are summarized as follows:

- A formal definition and formulation of the collision probability under multi-path transmission in multi-event-driven WSNs is presented, and then an energy efficiency maximization problem is formulated as a non-convex combinatorial fractional optimization problem under the constraints of maximum conflict probability, minimum success probability, and maximum transmission power.
- A dual-based iterative search algorithm with acceptable complexity is proposed to solve the optimization problem efficiently. To make it tractable, we employ the Dinkelbach transformation to convert the problem in fractional form into a problem in subtractive form, and then use the Lagrangian dual method in an iterative manner to obtain an approximate optimal power allocation and routing solution.
- Numerous results show that our proposed algorithm achieved a gain of 9.09% to 35.05% in energy efficiency and reduced network energy consumption by 17.09% to 39.12% compared to other routing algorithms. The advantage is attributed to the power control design that avoids excessive energy consumption on the one hand, and a controlled conflict probability that reduces unnecessary control overhead caused by multi-path conflicts and improves transmission concurrency on the other hand.

**Figure 1:**
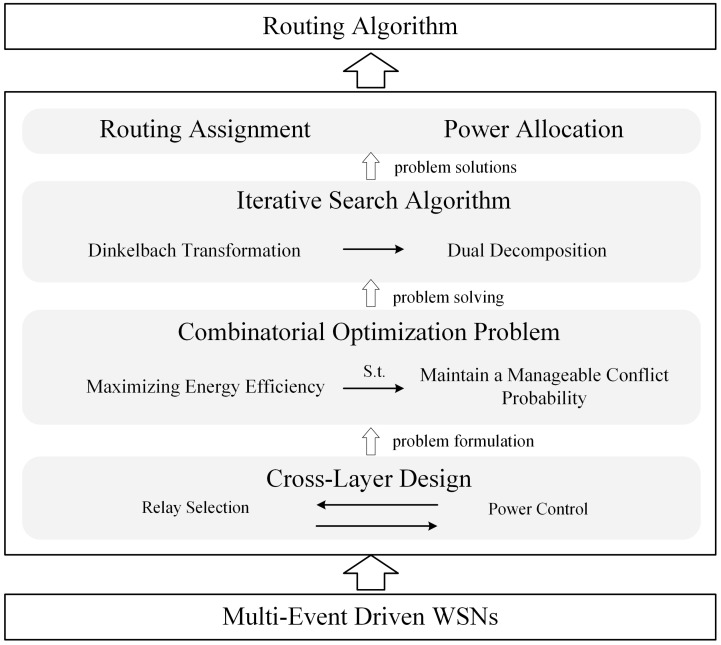
A sysytem overview of our cross-layer design.

A system overview of our cross-layer design is provided in Figure 1 to give the reader a complete impression of the algorithm.

## 2. Related Work

Reliable and energy-efficient transmission have been two of the most critical issues in the design of routing algorithms for wireless networks, especially for dense networks with strict energy constraints. In the following, we discuss related work and motivations from these two perspectives.

In recent years, many reliable routing algorithms have been proposed for wireless networks. The author in [16] proposed a cross-layer routing algorithm using fuzzy logic and topological control to reduce the control cost and improve routing reliability. The algorithm classified nodes into different classes based on their relay node degrees and adjusted their transmission power based on their classes. The source nodes determined their relay priority based on the utility of the candidate relay nodes, i.e., the relay node with the highest utility was responsible for forwarding the packet to the next hop. In [17], C. Xu et al. developed a packet reception probability model based on SINR, packet queue length, and link availability to describe the reliability of the links. Based on this, the routing decision problem was formulated as a constrained multi-objective optimization problem that attempted to find a link with the highest packet reception probability as a relay link. The author in [3] investigated how to construct minimum power routing with end-to-end reliability constraints. In [4], W. Yang et al. proposed an interruption-optimal relay selection algorithm based on a fuzzy comprehensive evaluation. By considering the channel state information and the collaboration power information of nodes, the optimal relay was selected using fuzzy-integrated judgment, thus minimizing the outage probability, i.e., maximizing the reliability of end-to-end transmission. F. Mansourkiaie et al. in [5] investigated a routing algorithm that used cooperative routing in WSNs to minimize the collision probability. The optimal route from each source to the aggregation node was established by providing the optimal set of relays in each route, the optimal set of hops, and the optimal power allocation for the transmission link. A hybrid K-means and logistic-regression-based algorithm was proposed in [18] for efficient and timely routing with minimal data loss.

In addition, for energy-constrained WSNs, the energy issue is also an essential research point in the design of routing algorithms. In [19], an energy-efficient optimal relay selection (EEORS) algorithm was proposed for underwater wireless networks, the core of which was to select relay nodes by combining the location and depth of the sensor nodes. The author in [12] achieved power consumption reduction via multi-hop short-range data communication. Numerical results in [10] verified that the multi-hop short-distance strategy can save up to 52% in power consumption. However, paths with a large number of relays imply low throughput. In [20], T. Kaur et al. proposed a QoS-aware cross-layer routing algorithm based on a multi-objective ant colony optimization. The algorithm considered the energy consumption and throughput as two optimization objectives to select the optimal routing path for event data transmission by using multi-pheromone information and multi-heuristic information consisting of two objective functions. In addition, many studies have typically employed power control techniques to reduce network energy consumption. In [21], a distributed energy-efficient and reliable routing algorithm with adaptive transmission power control was proposed by combining routing algorithm at the network layer and power control policy at the physical layer. A distributed routing algorithm based on optimal power allocation was proposed in [9] that achieved the minimum total transmit power from the source to the destination nodes. The author in [22] investigated an energy-efficient collaborative transmission problem and transformed it into a non-convex constrained optimization problem. Based on fractional programming and dual decomposition, a distributed iterative algorithm for power splitting, power allocation, and relay selection was developed to solve the optimization problem.

In general, most existing routing algorithms focus on data transmission on a single route, with little consideration given to multi-path transmission in multi-event-driven networks. Compared to previous routing algorithms, the design of cross-layer optimization for multi-path transmission is more challenging. On the one hand, there is the joint optimization of routing and power control to find the optimal transmission path and the optimal power allocation for the route. The decision processes are coupled with each other, which often leads to non-linear optimization and thus makes it difficult to provide the best solution. On the other hand, the conflict probabilities between multiple paths are difficult to evaluate systematically, and the high computational complexity and long computation times limit the applicability of the algorithms in practice due to the inherent difficulty of the problem. In this paper, we formally provide a mathematical characterization of the conflict probability in multi-path routing and propose an iterative dual-based search algorithm with acceptable complexity to efficiently solve the problem of maximizing energy efficiency with controlled conflict probability.

## 3. System Model

In this section, we first introduce the system description of the network architecture, transmission rate, and power consumption, and we then formalize the definition and expression of the collision probability in multi-path transmission.

### 3.1. System Description

**Figure 2:**
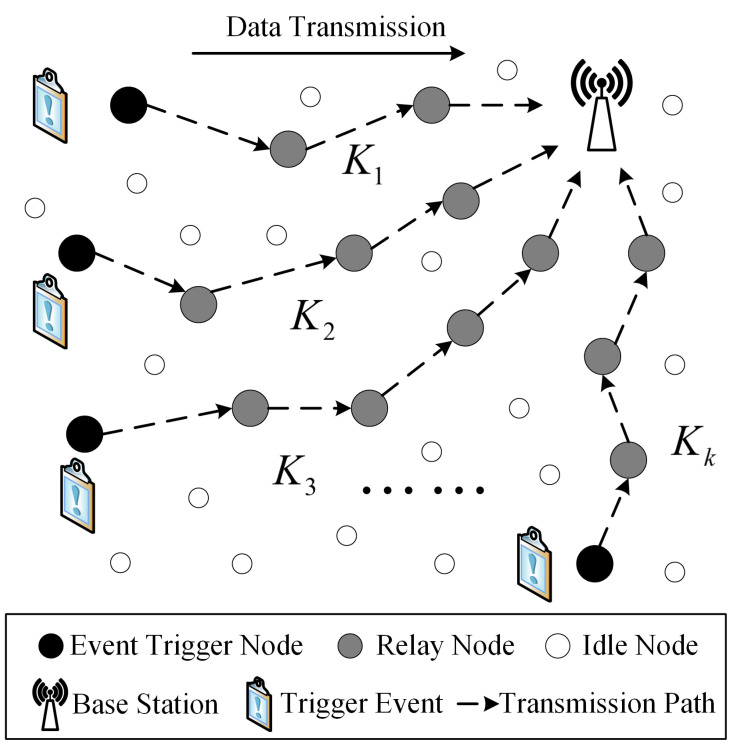
A topological structure of multi-hop transmission in a multi-event-driven network. The white circles represent the sensor nodes in an idle state; the black circles represent the event-driven nodes, which are responsible for generating event messages and forwarding them to the relay node; the gray circles represent relay nodes, which are responsible for forwarding event information to the base station.

We consider a tightly integrated event-driven wireless sensor network consisting of a BS and *N* deployed sensor nodes: $N_{1},N_{2},\cdots ,N_{n}$. The sensor nodes are triggered by different events to collect event information (e.g., temperature, pollution level, fire level, etc.) and send it to the BS via multi-hop transmission [23]. Assume that there are *K* routing paths, $K_{1},K_{2},\cdots ,K_{k}$, each of which contains multiple relay nodes. Figure 2 shows the transmission architecture of the network.

Similar to [24], we use a parameter $g_{i,j}$ to capture the loss of the signal power as the signal propagates from source node $N_{i}$ to destination node $N_{j}$ through the wireless channel. Therefore, the power received by $N_{j}$ from $N_{i}$ is $p_{i}g_{i,j}$, where $p_{i}$ denote the transmit power of $N_{i}$. Then, the transmission rate at $N_{j}$ for $N_{i}$ is expressed as follows [25]:

(1) $$R_{i}=B\times log\left(\frac{1+p_{i}g_{i,j}}{\sigma^{2}}\right),$$

where *B* represents the channel bandwidth, and $\sigma^{2}$ is the power level of the ambient noise.

Assume that each node is able to continuously adjust its transmission power from minimum to maximum. The consumed power for $N_{i}$ includes a practical allocated power and a fixed circuit power $P_{0}$ [26], which is given by the following:

(2) $$P_{i}=\frac{p_{i}}{\xi_{i}}+P_{0},$$

where $\xi_{i}$ represents a power amplifier coefficient of $N_{i}$.

For convenience, the parameters used in this paper are listed in Table 1.

### 3.2. Conflict Probability Model for Multi-Path Transmission

A CSMA-based MAC protocol is applied to eliminate contention and retransmission caused by conflicts. When a relay node accesses the channel, it may restrict the transmission of the relay nodes in other routing paths within the listening range. The probability of a relay node interfering with the relay nodes in other paths is defined as the multi-path conflict probability. Next, we provide the formal mathematical expression for the conflict probability.

**Figure 3:**
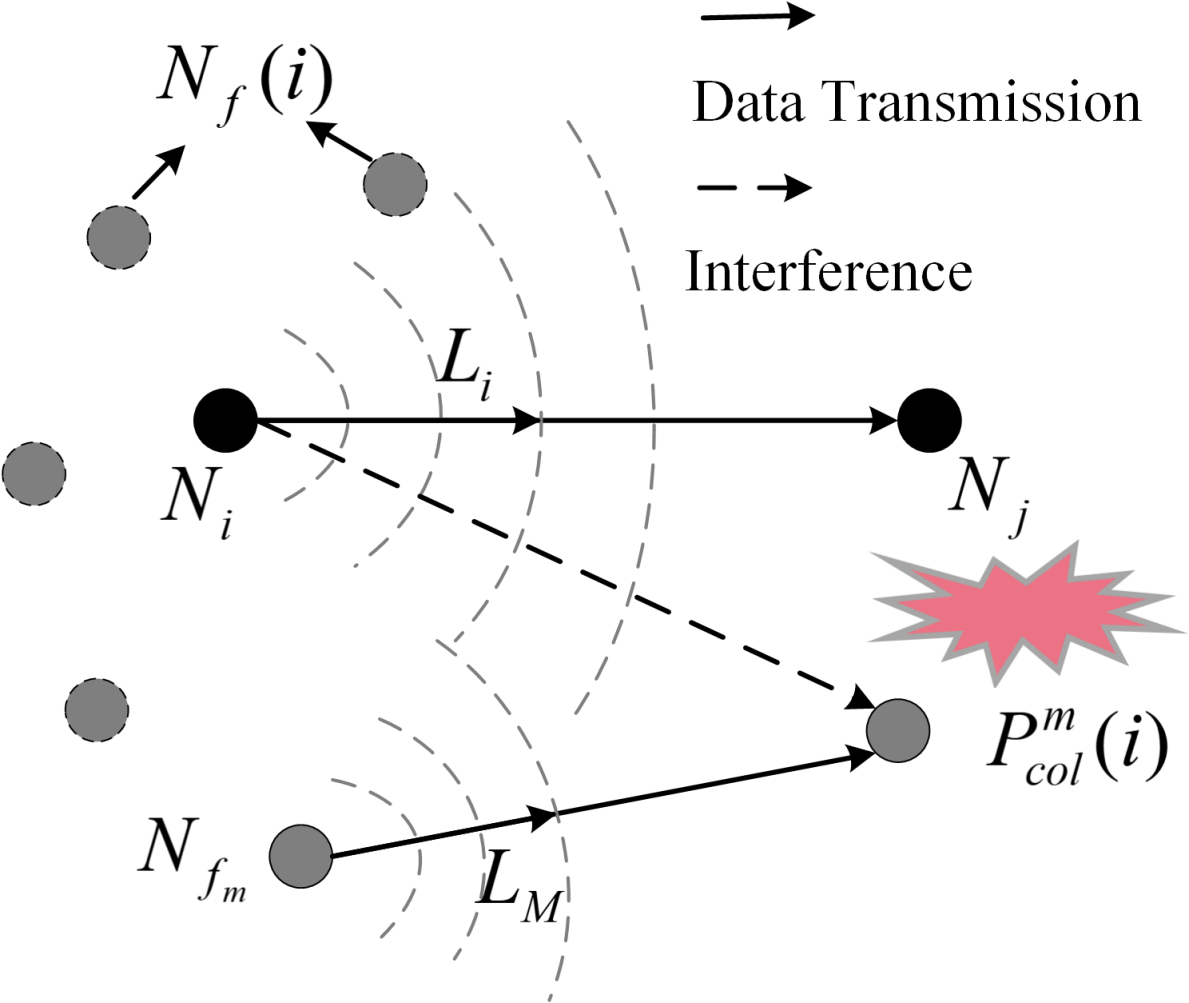
Multi-path conflict probability.

As shown in Figure 3, there are *M* interfering nodes $N_{f}\left(i\right)=\left\{N_{f_{1}},N_{f_{2}},\ldots ,N_{f_{M}}\right\}$ in the sensing area of the source node $N_{i}$. Denote $L_{f_{1}},L_{f_{2}},\ldots ,L_{f_{M}}$ and $L_{i}$ as the length of the data package to be transmitted by $N_{f_{1}},N_{f_{2}},\ldots ,N_{f_{M}}$ and $N_{i}$, respectively. The nodes within the transmitter’s listening range are not allowed to transmit while employing the carrier listen multiple access and conflict avoidance (CSMA-CA) technique for channel access. When $N_{i}$ and $N_{f_{m}},\forall m\in \left[1,M\right]$ broadcast data simultaneously, but $N_{i}$ is unaware of $N_{f_{m}}$, there is a conflict collision at the destination node of $N_{f_{m}}$.

Assuming that the arrival process of the data packet conforms to the Poisson distribution process, the probability density of the Poisson distribution is expressed as follows:

(3) $$P_{r}\left(D=x,\lambda T\right)=\frac{e^{-\lambda T}\times {\left(\lambda T\right)}^{x}}{x!},$$

where λ refers to the total number of data packets expected to be received within a specific duration *T*, and *x* represents the total number of data packets actually received in *T*.

Then, the transmission duration from $N_{f_{m}}$ to its relay node $N_{f_{n}}$ is calculated as follows:

(4) $$T_{\left(f_{m},f_{n}\right)}=\frac{L_{f_{m}}}{R_{f_{m}}},$$

where $R_{f_{m}}$ denotes the transmission rate of $N_{f_{m}}$.

During this period, the probability that $N_{f_{m}}$ receives one data packet from $N_{i}$ can be calculated as follows:

(5) $$P_{r}\left(D=1,L_{i}T_{\left(f_{m},f_{n}\right)}\right)=\exp\left(-L_{i}T_{\left(f_{m},f_{n}\right)}\right)\times L_{i}T_{\left(f_{m},f_{n}\right)}.$$

Thus, the probability that $N_{i}$ and $N_{f_{m}}$ have data transmission simultaneously is given by the following:

(6) $$\begin{array}{cc} p_{trans}^{m}\left(i\right) & =P_{r}\left(D\geq 1,L_{i}T_{\left(f_{m},f_{n}\right)}\right)\\ & =1-P_{r}\left(D=0,L_{i}T_{\left(f_{m},f_{n}\right)}\right)\\ & =1-\exp\left(-\frac{L_{i}L_{f_{m}}}{R_{f_{m}}}\right). \end{array}$$

Denote $p_{sens}^{m}\left(i\right)=p\left(I_{i,m}^{s}<I_{th}^{s}\right)$ as the perceived probability that $N_{i}$ cannot detect that $N_{f_{m}}$ is sending data at the same time, and $I_{th}^{s}$ is the carrier listening threshold, above which the channel is considered busy. $p_{inf}^{m}\left(i\right)=p\left(I_{i,m}^{f}<I_{th}^{f}\right)$ represents the probability that the signal strength sent from $N_{i}$ to the destination node of $N_{f_{m}}$ is greater than the interference threshold $I_{th}^{f}$. Assuming that the received signal is Rayleigh-distributed and that the signal-to-noise ratio is exponentially distributed [27], we have the following:

(7) $$p_{sens}^{m}\left(i\right)=1-\exp\left(-\frac{\sigma^{2}\left(2^{I_{th}^{s}}-1\right)}{p_{i}g_{i,m}}\right),$$

(8) $$p_{inf}^{m}\left(i\right)=\exp\left(-\frac{\sigma^{2}\left(2^{I_{th}^{f}}-1\right)}{p_{i}g_{i,n}}\right).$$

Thus, the probability of $N_{i}$ causing a conflict to $N_{f_{m}}$ is expressed as follows:

(9) $$\begin{array}{cc} P_{col}^{m}\left(i\right) & =p_{sens}^{m}\left(i\right)p_{inf}^{m}\left(i\right)p_{trans}^{m}\left(i\right)\\ & =\left(1-\exp\left(-\frac{\sigma^{2}\left(2^{I_{th}^{s}}-1\right)}{p_{i}g_{i,m}}\right)\right)\left(\exp\left(-\frac{\sigma^{2}\left(2^{I_{th}^{f}}-1\right)}{p_{i}g_{i,n}}\right)\right)\left(1-\exp\left(-\frac{L_{i}L_{f_{m}}}{R_{f_{m}}}\right)\right). \end{array}$$

Then, the probability of $N_{i}$ causing conflicts with $N_{f}\left(i\right)=\left\{N_{f_{1}},N_{f_{2}},\ldots ,N_{f_{M}}\right\}$ within its listening range can be obtained by the following:

(10) $$\begin{array}{c} P_{col}\left(i\right)=1-\prod_{m\in N_{f}\left(i\right)}\left(1-P_{col}^{m}\left(i\right)\right)\\ =\;1\;\;\;-\;\;\;\;\prod_{m\in N_{f}\left(i\right)}\left(\;1\;-\;\left(\;1\;-\;\exp\left(\;-\;\frac{\sigma^{2}\left(2^{I_{th}^{s}}\;-\;1\right)}{p_{i}g_{i,m}}\right)\right)\;\times \;\exp\left(\;-\;\frac{\sigma^{2}\left(2^{I_{th}^{f}}\;-\;1\right)}{p_{i}g_{i,n}}\right)\;\times \;\left(\;1\;-\;\exp\left(\;-\;\frac{L_{i}L_{f_{m}}}{R_{f_{m}}}\right)\right)\right). \end{array}$$

## 4. Problem Formulation of Energy Efficiency Maximization

In this section, we study an energy efficiency maximization problem under reliability constraints, and we provide a mathematical model for joint routing and power allocation.

Let N denote the set of sensor nodes and K denote the routing paths. We define a binary scheduling variable $\rho_{i}^{k}$ for node $N_{i}$ in routing path *k*.

(11) $$\rho_{i}^{k}=\left\{\begin{array}{cc} 1 & \;\mathrm{if}\;\mathrm{node}\;N_{i}\;\mathrm{is}\;\mathrm{assigned}\;\mathrm{to}\;\mathrm{path}\;k\;\\ 0 & \;\mathrm{otherwise}\; \end{array},\forall i\in N,\forall k\in K\right..$$

Since a node can only transmit data for a single event, i.e., it can only be assigned to one path, we have $\sum_{k\in K}\rho_{i}^{k}\leq 1,\forall i\in N$.

Denote $p_{i},\forall i\in N$ as the allocated power for $N_{i}$, and denote $g_{i,j},\forall i,j\in N$ as the channel gain for $N_{i}$ to its next-hop relay node $N_{j}$. Then, according to Equations (1) and (2), the achievable transmission rate of $N_{i}$ can be expressed as follows:

(12) $$R_{i}=\sum_{k\in K}\rho_{i}^{k}Blog\left(\frac{1+p_{i}g_{i,j}}{\sigma^{2}}\right),$$

and the consumed power for $N_{i}$ is can be defined as follows:

(13) $$P_{i}=\frac{\sum_{k\in K}\rho_{i}^{k}p_{i}}{\xi_{i}}+P_{0}.$$

**Definition** **1.** *(Energy efficiency): The system energy efficiency is defined as the total transmission rate $R_{tol}\left(\rho ,p\right)$ over the total transmission power $P_{tol}\left(\rho ,p\right)$, which is expressed as follows:*

(14) $$EE=\frac{R_{\mathrm{tol}}\left(\rho ,p\right)}{P_{\mathrm{tol}}\left(\rho ,p\right)}=\frac{\sum_{i\in N}\sum_{k\in K}\rho_{i}^{k}B\log\left(\frac{1+p_{i}g_{i,j}}{\sigma^{2}}\right)}{\sum_{i\in N}\sum_{k\in K}\rho_{i}^{k}p_{i}/\xi_{i}+P_{0}}.$$

Denote $N_{f}\left(i\right)$ as the interfering nodes in the sensing area of $N_{i}$. As discussed in Section 3.2, the probability of $N_{i}$ causing conflicts with $N_{f}\left(i\right)$ within its listening range is expressed as follows:

(15) $$\begin{array}{c} P_{col}\left(i\right)=1-\prod_{m\in N_{f}\left(i\right)}\left(1-P_{col}^{m}\left(i\right)\right)\\ =\;1\;\;\;-\;\;\;\;\prod_{m\in N_{f}\left(i\right)}\left(\;1\;-\;\left(\;1\;-\;\exp\left(\;-\;\frac{\sigma^{2}\left(2^{I_{th}^{s}}\;-\;1\right)}{p_{i}g_{i,m}}\right)\right)\;\times \;\exp\left(\;-\;\frac{\sigma^{2}\left(2^{I_{th}^{f}}\;-\;1\right)}{p_{i}g_{i,n}}\right)\;\times \;\left(\;1\;-\;\exp\left(\;-\;\frac{L_{i}L_{f_{m}}}{R_{f_{m}}}\right)\right)\right). \end{array}$$

Similarly, $P_{sucs}\left(i\right)=P\left(I_{i,j}>I_{th}^{s}\right)$ represents the probability of $N_{i}$ successfully transmitting to $N_{j}$, which is specifically expressed as follows:

(16) $$P_{sucs}\left(i\right)=exp\left(-\frac{\sigma^{2}\left(2^{I_{th}^{s}}-1\right)}{p_{i}g_{i,j}}\right).$$

In order to construct a reliable and energy-efficient transmission path, we designed a cross-layer optimization model to find the optimal routing policy ρ and power allocation policy *p* with the objective of maximizing energy efficiency under reliability constraints. The optimization problem can be formulated as follows:

(17) $$\begin{array}{c} \underset{\rho ,p}{Max}EE\\ s.t.\\ C1:\rho_{i}^{k}=\left\{0,1\right\},\forall i\in N,\forall k\in K\\ C2:\sum_{k\in K}\rho_{i}^{k}\leq 1,\forall i\in N\\ C3:\sum_{k\in K}\rho_{i}^{k}P_{col}\left(i\right)<\theta_{\alpha },\forall i\in N\\ C4:\sum_{k\in K}\rho_{i}^{k}P_{sucs}\left(i\right)\geq \theta_{\beta },\forall i\in N\\ C5:0\leq p_{i}\leq P_{max},\forall i\in N. \end{array}$$

Constraint C1 defines the binary scheduling variable, and Constraint C2 reveals that each node can only undertake the forwarding task of at most one event. Constraint C3 indicates that the probability of the multi-path conflict is not greater than the conflict probability threshold $\theta_{\alpha }$. Constraint C4 is for ensuring the success transmission probability of the routing path, where $\theta_{\beta }$ is the target end-to-end success probability. The transmission power of each node is indicated by Constraint C5.

## 5. Dual-Based Iterative Search Algorithm for Energy Efficiency Maximization Problem

In this section, we propose an iterative search algorithm that uses non-linear fractional programming and Lagrange dual theory to obtain the approximate optimal solution of the energy efficiency maximization problem.

### 5.1. Dinkelbach Transformation

First, using non-linear fractional programming, the objective function in fractional form is converted into an equivalent objective function in subtractive form [22,25].

**Theorem** **1.** *Denote a maximum weighted energy efficiency as follows:*

(18) $$q^{*}=\underset{(\rho ,p)\in R}{Max}\frac{R_{\mathrm{tol}\;}\left(\rho ,p\right)}{P_{\mathrm{tol}\;}\left(\rho ,p\right)}.$$

*where $R=\left\{\left(\rho ,p\right)|\sum_{i\in N}\rho_{i}^{k}\leq 1,\rho_{i}^{k}=\left\{0,1\right\},\forall i\in N,k\in K;0\leq p_{i}\leq P_{\max},\forall i\in N\right\}$. If and only if*

(19) $$U\left(q^{*}\right)=\underset{(\rho ,p)\in R}{Max}\left\{R_{\mathrm{tol}\;}\left(\rho ,p\right)-q^{*}P_{\mathrm{tol}\;}\left(\rho ,p\right)\right\}=0.$$

The maximum energy efficiency $q^{*}$ is achieved by the optimal routing and power allocation policies (ρ,p).

Theorem 1 reveals that the fractional programming problem (17) can be transformed into an equivalent subtractive-form optimization problem (19). Dinkelbach provides an iterative algorithm for solving this optimization problem [25]. Specifically, in each iteration, for a given parameter *q*, the transformed maximization problem (19) is solved by dual decomposition and obtains the alternative optimal routing and power allocation policies (ρ,p). Then, update the *q* and use it to solve the maximization problem (19) in the next iteration until it reaches the maximal iterations or until the condition $R_{tol}\left(\rho ,p\right)-qP_{tol}\left(\rho ,p\right)<\varepsilon$ is satisfied. The transformed problem for *q* can be expressed as follows:

(20) $$\begin{array}{c} \underset{(\rho ,p)\in R}{Max}\left\{R_{tol}\left(\rho ,p\right)-qP_{tol}\left(\rho ,p\right)\right\},\\ s.t.\\ C3-C4. \end{array}$$

Since the dual variable $\rho_{i}^{k}\in \left\{0,1\right\}$, the problem remains a mixed combinatorial optimization problem, which is typically non-convex and NP-hard. The literature [22] has proven the zero duality gap between the problem (20) and its dual problem, which implies that the optimal solution of the dual problem is also the solution of the original problem. Therefore, we next use the Langerian dual method to solve the problem (20).

### 5.2. Dual Problem Formulation

According to the Taylor formula, we have $\lim_{x\to 0}\left(exp\left(-x\right)\right)=1-x$ and $\lim_{x\to 0}\left(1-exp\left(-x\right)\right)=x$. By using a first-order Taylor polynomial approximation on Equation (15), Equation (15) can be simplified to the following:

(21) $$P_{col}\left(i\right)=1-\prod_{m\in N_{f}\left(i\right)}\left(1-\left(\left(1-\frac{\sigma^{2}\left(2^{I_{th}^{f}}-1\right)}{p_{i}g_{i,n}}\right)\times \frac{\sigma^{2}\left(2^{I_{th}^{s}}-1\right)}{p_{i}g_{i,m}}\times \frac{L_{i}L_{f_{m}}}{R_{f_{m}}}\right)\right).$$

Then, Constraint C3 can be written as follows:

(22) $$\varphi \left(p_{i}\right)=\sum_{k\in K}\rho_{i}^{k}\left(1-\prod_{m\in N_{f}\left(i\right)}\left(1-\left(\left(1-\frac{\sigma^{2}\left(2^{I_{th}^{f}}-1\right)}{p_{i}g_{i,n}}\right)\times \frac{\sigma^{2}\left(2^{I_{th}^{s}}-1\right)}{p_{i}g_{i,m}}\times \frac{L_{i}L_{f_{m}}}{R_{f_{m}}}\right)\right)\right)-\theta_{\alpha }\leq 0.$$

Similarly, $P_{sucs}\left(i\right)$ can be expressed as $1-\sigma^{2}\left(2^{I_{th}^{s}}-1\right)/p_{i}g_{i,j}$, and we have the following:

(23) $$\eta \left(p_{i}\right)=\sum_{k\in K}\rho_{i}^{k}\left(1-\frac{\sigma^{2}\left(2^{I_{th}^{s}}-1\right)}{p_{i}g_{i,j}}\right)-\theta_{\beta }\geq 0.$$

Then, we provide the Lagrangian function of problem (20) via the following:

(24) $$\begin{array}{c} L\left(\rho ,p,\lambda ,\mu \right)=\sum_{i\in N}\sum_{k\in K}\rho_{i}^{k}B\log\left(1+\frac{p_{i}g_{i,j}}{\sigma^{2}}\right)-q\left(\sum_{i\in N}\sum_{k\in K}\frac{\rho_{i}^{k}p_{i}}{\xi_{i}}+P_{0}\right)\\ +\sum_{i\in N}\lambda_{i}\left(\theta_{\alpha }+\sum_{k\in K}\rho_{i}^{k}\left({\left(1-\frac{\psi (i)}{p_{i}^{2}}\right)}^{\left|N_{f}\left(i\right)\right|}-1\right)+\sum_{i\in N}\mu_{i}\left(\sum_{k\in K}\rho_{i}^{k}\left(1-\frac{\omega (i)}{p_{i}}\right)-\theta_{\beta }\right)\right., \end{array}$$

where $\psi \left(i\right)=\sum_{m\in N_{f}\left(i\right)}\frac{\sigma^{2}\left(2^{I_{th}^{f}}-1\right)}{g_{i,n}}\frac{\sigma^{2}\left(2^{I_{th}^{s}}-1\right)}{g_{i,m}}\frac{L_{i}L_{f_{m}}}{R_{f_{m}}}/\left|N_{f}\left(i\right)\right|$, and $\omega \left(i\right)=\frac{\sigma^{2}\left(2^{I_{th}^{s}}-1\right)}{g_{i,j}}$. $\lambda =\left\{\lambda_{1},\lambda_{2},\ldots ,\lambda_{N}\right\}$, and $\mu =\left\{\mu_{1},\mu_{2},\ldots ,\mu_{N}\right\}$ are the Lagrange multipliers corresponding to the maximum conflict probability of Constraint C3 and the minimum success probability of Constraint C4, respectively.

According to Equation (24), the dual function $h\left(\rho ,p,\lambda ,\mu \right)$ can be formulated as follows:

(25) $$h\left(\rho ,p,\lambda ,\mu \right)=\min_{\lambda ,\mu }\max_{\rho ,p}L\left(\rho ,p,\lambda ,\mu \right).$$

**Figure 4:**
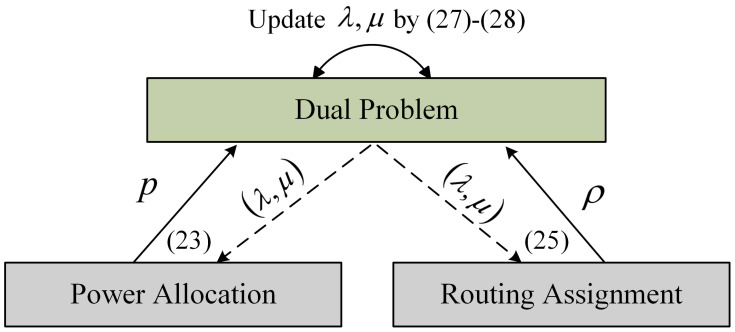
The iterative process of solving dual problem, where the numbers represent the equation numbers.

Note that the solution to Equation (20) can be derived from the dual problem (25). Next, we apply an iterative approach to solve the dual problem. By employing the KKT conditions, we first optimize the Lagrangian over the primal variables (ρ,p) given the dyadic variables (λ,μ). Then, using the primal variables (ρ,p), we update the dyadic variables (λ,μ) using the subgradient method. Figure 4 shows the iterative process, the details of which will be illustrated in the following section.

### 5.3. Power Allocation and Routing Assignment

Given λ and μ, the optimal $p_{i}$ can be obtained through KKT conditions. By taking the derivative of L(ρ,p,λ,μ) with respect to $p_{i}$ to zero and solving the following equations, we have the following:

(26) $$\left\{\begin{array}{c} \frac{\partial L(\rho ,p,\lambda ,\mu )}{\partial p}=0\;\\ \varphi (p)=0\;\\ \eta (p)=0. \end{array}\right.$$

Then, the optimal power allocation of $N_{i}$ is obtained as follows:

(27) $$p_{i}^{*}={\left[\frac{g_{i,j}-\chi \left(i\right)\sigma^{2}}{g_{i,j}\xi_{i}\left(\chi \left(i\right)+B/In2\right)}\right]}_{0}^{P_{\max}},$$

where $\chi \left(i\right)=\mu_{i}\left(1-\theta_{\beta }\right)+2\lambda_{i}\left|N_{f}\left(i\right)\right|\left(1-\theta_{\alpha }\right)$; ${\left[x\right]}_{a}^{b}$ is defined as ${\left[x\right]}_{a}^{b}=max\left(a,min\left(x,b\right)\right)$.

With the fixed variables $p_{i}$, $\mu_{i}$, $\lambda_{i}$, the Lagrangian function L(ρ,p,λ,μ) is linear with respect to $\rho_{i}^{k}$ and falls in the interval $\left\{0,1\right\}$. Hence, the maximum value of L(ρ,p,λ,μ) can be divided into two situations: (1) if ∂L(ρ,p,λ,μ)/∂ρ>0, then the maximum value can be obtained via $\rho_{i}^{k}=1$; and (2) if ∂L(ρ,p,λ,μ)/∂ρ<0, then the maximum value can be obtained via $\rho_{i}^{k}=0$. That is,

(28) $$\frac{\partial L\left(\rho_{i}^{k},p_{i},\lambda_{i},\mu_{i}\right)}{\partial \rho_{i}^{k}}=B\log\left(1\;+\;\frac{p_{i}^{*}g_{i,j}}{\sigma^{2}}\right)\;-\;\frac{qp_{i}^{*}}{\xi_{i}}\;+\;\lambda_{i}\left({\left(1\;-\;\frac{\psi (i)}{p_{i}^{*2}}\right)}^{\left|N_{f}\left(i\right)\right|}\;-\;1\right)+\mu_{i}\left(1\;-\;\frac{\omega (i)}{p_{i}^{*}}\right).$$

Define $M_{i}^{k}=\partial L\left(\rho_{i}^{k},p_{i},\lambda_{i},\mu_{i}\right)/\partial \rho_{i}^{k}$, which can be regarded as the marginal benefit of assigning $N_{i}$ to path *k*. Therefore, we allocate the $N_{i}$ with the largest $M_{i}^{k}$ as a relay node to path *k*. $\rho_{i}^{k}$ satisfies the following as a result:

(29) $$\rho_{i}^{k}=\left\{\begin{array}{cc} 1 & \;\mathrm{if}\;i=\arg\max_{i=1,\cdots ,N}M_{i}^{k}\;\mathrm{and}\;M_{i}^{k}>0\\ 0 & \;\mathrm{otherwise}\;. \end{array}\right.$$

### 5.4. Dual Problem Optimization

Given λ and μ, Equations (27) and (29) provide the optimal routing and power allocation policies $\rho^{*},p^{*}$. By substituting $\rho^{*},p^{*}$ into Equation (25), we can obtain the function of the dual problem as the following:

(30) $$\begin{array}{c} \min_{\lambda ,\mu }h\left(\lambda ,\mu \right),\\ s.t.\\ \lambda_{i}\geq 0,\mu_{i}\geq 0. \end{array}$$

Since the dyadic problem is, by definition, always a convex optimization problem, the commonly used gradient descent and ellipsoidal methods can be used to update the dyadic variables and converge towards a globally convergent optimal solution. In this paper, we used the gradient descent method to obtain the optimal values of λ and μ.

Specifically, the Lagrange multipliers update with the following equations:

(31) $$\lambda_{i}\left(t+1\right)={\left[\lambda_{i}\left(t+1\right)-\Delta \tau_{1}\left(t\right)\left(\theta_{\alpha }+\sum_{k\in K}\rho_{i}^{k}\left({\left(1-\frac{\psi_{f}\left(i\right)}{p_{i}^{2}}\right)}^{\left|N(i)\right|}-1\right)\right)\right]}^{+},$$

(32) $$\mu_{i}\left(t+1\right)={\left[\mu_{i}\left(t+1\right)-\Delta \tau_{2}\left(t\right)\left(\theta_{\beta }+\left(\sum_{k\in K}\rho_{i}^{k}\left(1-\frac{\omega (i)}{p_{i}}\right)-\theta_{\beta }\right)\right)\right]}^{+},$$

where *t* is an iteration index, and $\tau_{r}\left(t\right)$ is positive diminishing step size, which satisfies the following:

(33) $$\sum_{t=1}^{\infty }\tau_{r}\left(t\right)=\infty ,\lim_{t\to \infty }\tau_{r}\left(t\right)=0,\forall r\in \left\{1,2\right\}.$$

At each iteration, the Lagrange multiplier λ,μ is adjusted by Equations (31) and (32). The variables ρ,p of Equations (27) and (29) are then adjusted by using the updated Lagrange multipliers. The process is repeated until convergence is achieved or until the maximum number of iterations is reached. Algorithm 1 outlines the pseudo-code of the entire process.
**Algorithm 1** Dual-based iterative search algorithm for energy efficiency maximization problem1:**Require**:2:ε: an infinitesimal number;3:$iter_{max}$: maximum number of iterations;4:q←0: energy efficiency;5:t←1: iterative index;6:**Repeat**7:Initialize $\lambda_{i}\left(t\right)$, $\mu_{i}\left(t\right)$;8:   **Repeat**9:      Obtain $\rho_{i}^{*},p_{i}^{*}$ using Equations (27) and (29) with given $\lambda_{i}\left(t\right)$, $\mu_{i}\left(t\right)$ and *q*.10:      Update Lagrange multiplier $\lambda_{i}\left(t\right)$ and $\mu_{i}\left(t\right)$ by Equations (31) and (32);11:   **Until** λ and μ converge;12:**if** $R_{tol}\left(\rho^{*},p^{*}\right)-qP_{tol}\left(\rho^{*},p^{*}\right)<\varepsilon$ or $t<iter_{max}$ **then**13:    t←t+1;14:    Update $q=\frac{R_{tol}\left(\rho ,p\right)}{P_{tol}\left(\rho ,p\right)}$;15:**else**16:    $p^{opt}=p^{*}$;17:    $\rho^{opt}=\rho^{*}$;18:    $EE^{opt}=q^{*}$;19:    **Return**;20:**end if**21:**End**;

## 6. Simulation Results

In this section, the effectiveness of our proposed algorithm has been evaluated through extensive simulations in Matlab.

### 6.1. Simulation Setup

We applied the dataset generation framework in [28] to randomly deploy 50∼150 sensor nodes and 5∼20 event trigger nodes in a 100 × 100 m${}^{2}$ area. The initial energy and maximum transmission power of the sensor nodes were assumed to be equal and set to 0.1 J and 0.1 W, respectively. The listening area range of each node was similarly fixed at 20 m. In order to characterize different types of event triggers, it was assumed that different trigger nodes generated packets of different sizes from 10 to 100 (bit/ms) in each time interval and forwarded them to a centrally located BS in a multi-hop manner. We assumed the channel bandwidth (*B*) equaled 200 KHZ, and the power of ambient noise ($\sigma^{2}$) equaled −90 dBm. Referring to [5], we assumed that the carrier listening threshold ($I_{th}^{s}$) and interference threshold ($I_{th}^{f}$) were equal to the power of the ambient noise, $I_{th}^{s}=I_{th}^{f}=\sigma^{2}$. The conflict probability threshold ($\theta_{\alpha }$) and the success probability threshold ($\theta_{\beta }$) were set to 0.5 and 0.85, respectively. For the iterative search algorithm, the maximum number of iterations ($iter_{max}$) was defined to be 20. ε is a small value used to control the convergence of the algorithm, which was set to 0.001. The considered simulation parameters were cited from [5,21,28] and are given in Table 2.

### 6.2. Performance Analysis

**Figure 5:**
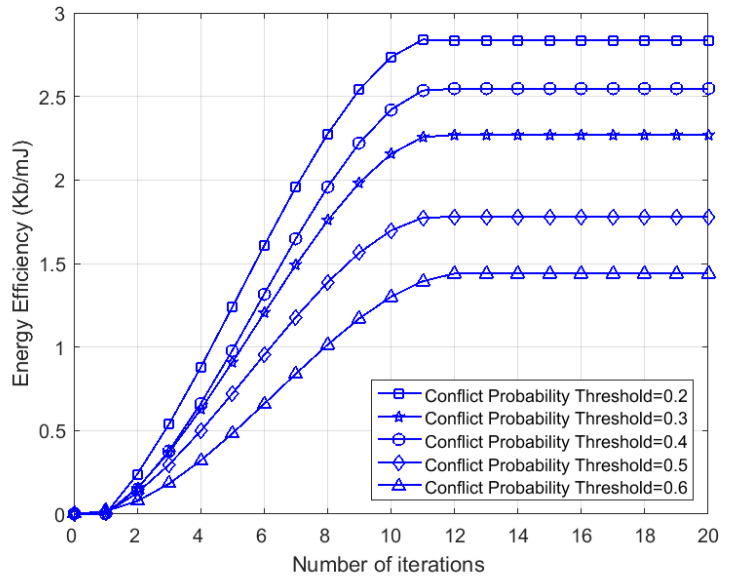
Evolution of energy efficiency with the number of iterations under conflict probability threshold $\theta_{\alpha }=\left\{0.2,0.3,0.4,0.5,0.6\right\}$ (K=10,N=100).

To begin with, we studied the convergence of our proposed algorithm, and Figure 5 depicts the evolution of the energy efficiency with the number of iterations under the conflict probability threshold $\theta_{\alpha }=\left\{0.2,0.3,0.4,0.5,0.6\right\}$. It is apparent that the energy efficiency increased with the number of iterations and converged within 11 iterations in all situations. It is also observed that a larger $\theta_{\alpha }$ led to lower network energy efficiency. In MINLP, the conflict probability is a constraint function during routing, which requires that the conflict probability caused by either routing path cannot be greater than a threshold $\theta_{\alpha }$. An increase in $\theta_{\alpha }$ implies that more strongly interfering relay nodes are allowed, which result in more collision conflicts when nodes transmit. As a result, the energy efficiency gradually decreases due to the loss of throughput caused by interference avoidance and the loss of energy consumption caused by control messages (e.g., RTS/CTS).

**Figure 6:**
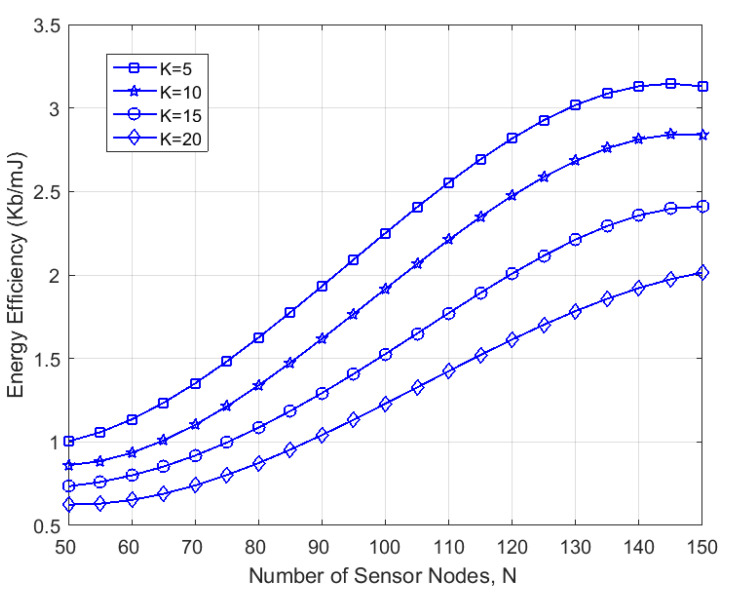
Variation in energy efficiency for different number of trigger events *K* as they varied with the number of sensor nodes *N*.

Next, we verified the energy efficiency of the proposed algorithm with respect to the number of trigger event nodes *K* and the number of sensor nodes *N*. Figure 6 depicts how the energy efficiency varied with *N* for $K=\left\{5,10,15,20\right\}$. As we can observe, the energy efficiency increased with the increase in *N*. This is because, on the one hand, the more nodes that were deployed, the shorter the average distance between two neighboring nodes and, therefore, the lower the transmission power required. On the other hand, in denser networks, nodes have more neighbors and, therefore, more options for data transmission, thus resulting in better energy efficiency performance. However, as *N* increased further, the increase in energy efficiency became slower and eventually stabilized. This was mainly due to the shortening of the transmission distance, which caused more collision delays and the energy consumption of control messages. Also, it can be observed that the energy efficiency was inversely proportional to *K*. Similarly, an increase in the number of trigger events undoubtedly caused more collisions. This not only reduced the transmission concurrency, but also caused more control overhead, which led to a degradation of the energy efficiency performance.

### 6.3. Performance Comparison

In this section, we compared the performance of our proposed algorithm with several existing routing algorithms, such as the DPRC algorithm presented in [21], the TOR algorithm presented in [29], and the MACO-QCR algorithm presented in [20]:

- DPRC (an energy-efficient routing algorithm with adaptive transmission power control): DPRC uses cross-layer design techniques that integrate network layer routing and physical layer power control strategies to construct minimum power routing with end-to-end reliability constraints;
- TOR (a throughput-optimal routing algorithm): TOR analyses the given relationship between the throughput and distance and determines the physical location of the relay to achieve the optimum MAC throughput from the trigger node to the target BS;
- MACO-QCR (A QoS-aware cross-layer routing algorithm based on a multi-objective ant colony optimization): MACO-QCR considers energy consumption and throughput as two optimization objectives to select the optimal routing path for event data transmission by using multi-pheromone information and multi-heuristic information consisting of two objective functions.

In general, all of the above comparison algorithms are proposed routing algorithms for multi-hop transmission in WSNs.

**Figure 7:**
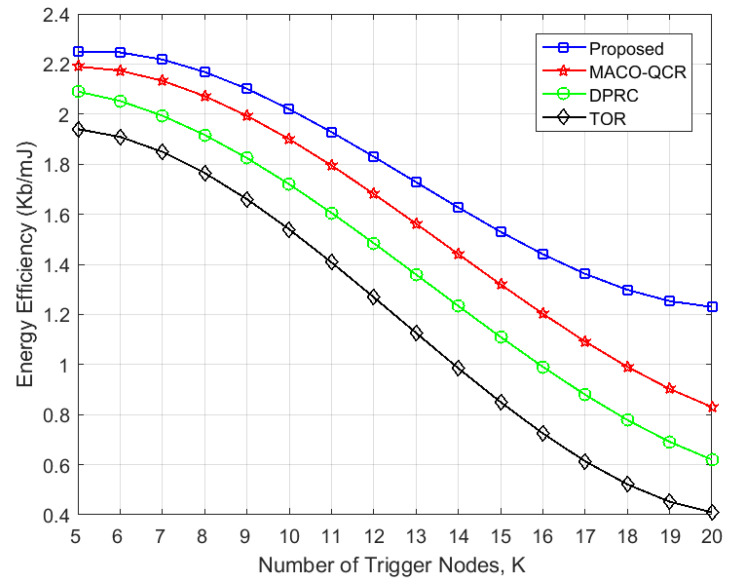
Variation in energy efficiency with the number of trigger events *K* (*N* = 100).

First, Figure 7 presents a comparison of the energy efficiency that varied with the number of trigger event nodes. It shows that our proposed algorithm exhibited much better performance regarding energy efficiency than the other algorithms and was 20.52% higher in efficiency than the DPRC, 35.05% higher than the TOR, and 9.09% higher than the MACO-QCR. Clearly, the TOR had the lowest energy efficiency, which is due to the absence of a power control mechanism, thus resulting in unnecessary energy consumption. The DPRC and MACO-QCR utilize power control to lower the energy consumption of the network, and the MACO-QCR further trades off energy consumption and throughput in its routing design. Both algorithms are effective in improving the energy efficiency of the network to a certain extent; however, they do not take into account the problem of interference collisions between nodes. As the number of trigger events increases, collisions between relay nodes not only introduce more energy consumption in control messages, but also reduce transmission concurrency, thus resulting in throughput degradation. Conversely, our algorithm maximized energy efficiency under the constraint of minimizing collision probability, which can effectively improve energy efficiency while accommodating interference collisions in multi-path transmission.

**Figure 8:**
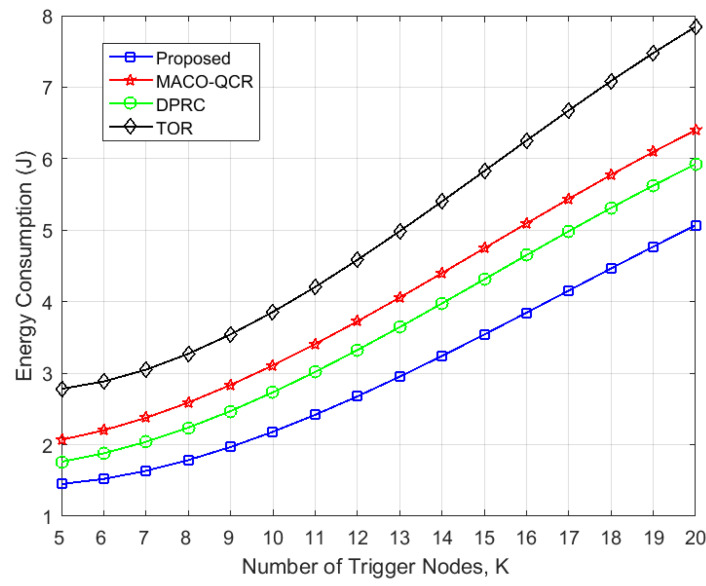
Variation in energy consumption with the number of trigger events *K* (*N* = 100).

In Figure 8, we compare the energy consumption for all algorithms in the same network scenario as in Figure 7. It can be observed that our algorithm had the lowest energy consumption, followed by the DPRC, the MACO-QCR, and the TOR. Specifically, the energy consumption of our algorithm was reduced by 17.09%, 24.84%, and 39.12% compared with the DPRC, MACO-QCR, and TOR, respectively. The TOR, as a throughput optimization method, always obtains the highest throughput at the expense of energy consumption, while the energy-efficiency-oriented DPRC and MACO-QCR algorithms have significant consumed control overhead during interference avoidance in multi-event triggered scenarios, as they do not consider interference conflicts for multi-path transmission. In contrast, our algorithm provides a mathematical characterization of the conflict probability under multi-path transmission and combines routing and power control to reduce the conflict probability of multi-path transmission, thus effectively reducing the network energy consumption.

**Figure 9:**
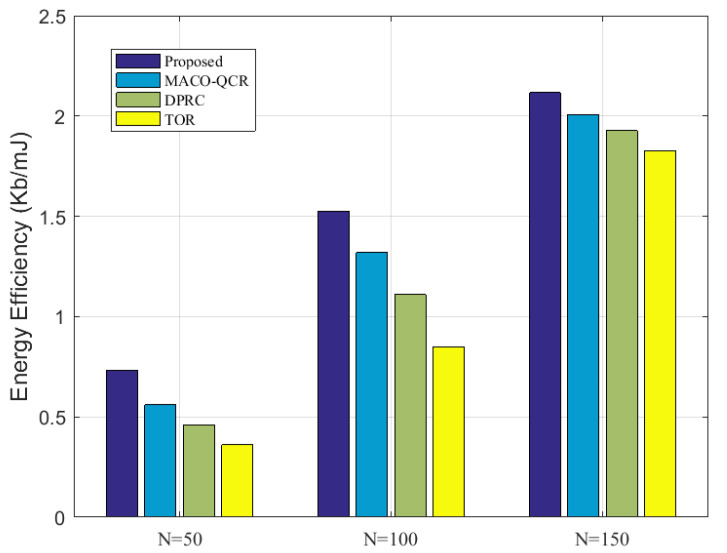
Comparison of energy efficiency at $N=\left\{50,100,150\right\}$ with *K* = 15.

Next, we further compared the energy efficiency of all the algorithms at different network densities. From Figure 9, we can observe that the energy efficiency was highest at *N* = 150, and smallest at *N* = 50, which is consistent with the results in Figure 6. With the number of sensor nodes from 50 to 150, our algorithm outperformed the MACO-QCR by about 9.68%, the DPRC by about 18.48%, and the TOR by about 29.55% on average. It can be seen that our proposed algorithm achieved better performance than other algorithms in both dense and sparse networks. This further illustrates the advantage of our algorithm in terms of energy efficiency.

**Figure 10:**
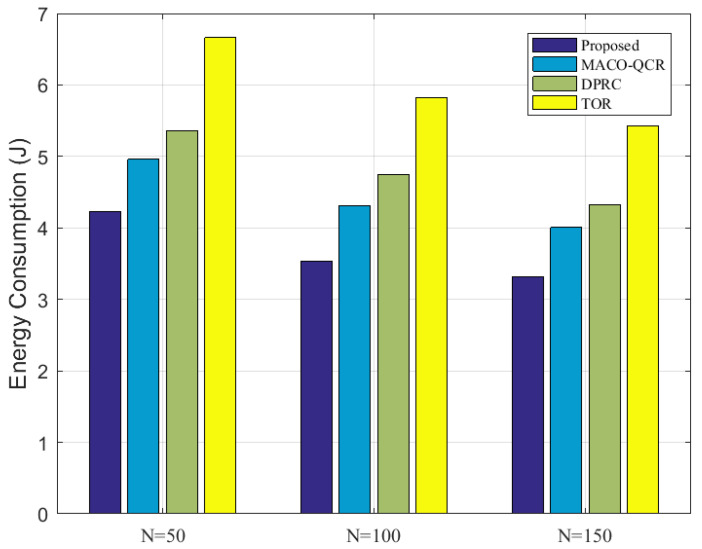
Comparison of energy losses at $N=\left\{50,100,150\right\}$ with *K* = 15.

Finally, Figure 10 compares the energy losses of all the algorithms for different network densities. Similarly, it can be seen that better transmission performance could be produced in denser networks due to the greater number of data transmission options available to the nodes. As the network density increased, the energy consumption of our algorithm decreased from 4.23 J to 3.31 J, which was less than that of the MACO-QCR, DPRC, and TOR algorithms. In particular, the average energy consumption of our algorithm with N=50,100,150 reduced by about 16.37% compared to the MACO-QCR, by about 23.06% compared to the DPRC, and by about 37.99% compared to the TOR. This comparison proves that our algorithms can effectively reduce the transmission energy consumption of the network and thus extend the lifetime of the network.

## 7. Conclusions

In this paper, we addressed the joint routing and power allocation problem from a cross-layer design perspective, thereby effectively maximizing the energy efficiency of the network within an acceptable probability of collision. First, the collision problem for multi-path transmission was defined and formulated formally. Then, the joint optimization problem was formulated as a mixed integer and non-linear programming problem, thereby enabling multi-path transmission to maximize energy efficiency and to satisfy the practical requirements of low conflict probability and high reliability. To solve this NP-hard problem, we provided a near-optimal solution for power allocation and routing using non-linear fractional programming and dual decomposition. Numerous results demonstrated that the routing and power control design enabled our proposed algorithm to achieve 39.12% lower energy consumption than the existing routing algorithms. In addition, our algorithm effectively avoided excessive energy consumption and reduced unnecessary control overhead caused by multi-path conflicts, thus achieving a 9.09% to 35.05% improvement in energy efficiency.

Our algorithm is a centralized algorithm that requires global information to manage multi-path transmission in event-driven WSNs. In the follow-up research, we will consider expanding a distributed routing algorithm to maximize energy efficiency while avoiding multi-path conflicts.

## Figures and Tables

**Table 1:** Notations and definitions.

$N=\left\{N_{1},N_{2},\cdots ,N_{n}\right\}$	Node set, where $N_{1},N_{2},\cdots ,N_{n}$ represent the randomly distributed sensor nodes in a event-driven WSN.
$K=\left\{K_{1},K_{2},\cdots ,K_{k}\right\}$	Path set, where $K_{1},K_{2},\cdots ,K_{k}$ represent the routing paths from the k-th event triggered node to the BS, respectively.
$N_{f}\left(i\right)\;=\;\left\{\;N_{f_{1}},\cdots ,N_{f_{M}}\;\right\}$	Interference set, where $N_{f_{1}},N_{f_{2}},\cdots ,N_{f_{M}}$ represent the interference nodes int the sensing area of $N_{i}$.
$g_{i,j}$	Channel gains, used to capture the loss of signal power as the signal propagates from the source node $N_{i}$ to the destination node $N_{j}$.
$p_{i}$	The transmission power of node $N_{i}$ during data transmission.
$p_{max}$	The maximum transmission power allowed for nodes during data transmission.
*B*	The channel bandwidth.
$\sigma^{2}$	The power level of the ambient noise.
$L_{i}$	The length of the data package to be transmitted by $N_{i}$.
$\xi_{i}$	The power amplifier coefficient of $N_{i}$.
$I_{th}^{s}$	Carrier listening threshold, beyond which the channel is considered busy.
$I_{th}^{f}$	Interference threshold, below which the interfering node does not affect the signal reception of the destination node.
$\rho_{i}^{k}$	Binary scheduling variable: $\rho_{i}^{k}=1$ represents that $N_{i}$ is assigned to routing path $K_{k}$, otherwise $\rho_{i}^{k}=0$.
$\theta_{\alpha }$	Conflict probability threshold that is the maximum allowed conflict probability of the routing path.
$\theta_{\beta }$	Success probability threshold that is the minimum probability of successful transmission expected by the routing path.
$\lambda =\left\{\lambda_{1},\lambda_{2},\ldots ,\lambda_{N}\right\}$	Lagrange multipliers corresponding to the maximum conflict probability constraint.
$\mu =\left\{\mu_{1},\mu_{2},\ldots ,\mu_{N}\right\}$	Lagrange multipliers corresponding to the minimum success probability constraint.
$\tau_{r}\left(t\right)$	Positive diminishing step size, where *t* is an iteration index
ε	Convergence coefficient, which is a small value that controls the convergence of the algorithm.
$Iter_{max}$	Maximum number of iterations, which is used to control the stopping of the iterative algorithm.

**Table 2:** Simulation parameters.

Parameter	Value	Parameter	Value
Number of sensor nodes	100	Number of event trigger nodes	10
Listening area range	20 (m)	Packet generation rate	10∼100 (bit/ms)
Initial energy	0.1 (J)	Maximum transmission power	0.1W
Carrier listening threshold	−90 (dBm)	interference threshold	−90 (dBm)
Conflict probability threshold	0.5	Success probability threshold	0.85
Channel bandwidth	200 (KHZ)	Power of ambient noise	−90 (dBm)
Maximum number of iterations	20	Convergence coefficient	0.001

## Data Availability

Not applicable.
